# Thoracic injuries in trauma patients: epidemiology and its influence on mortality

**DOI:** 10.1186/s13049-022-01058-6

**Published:** 2022-12-12

**Authors:** Andrea Lundin, Shahzad K. Akram, Lena Berg, Katarina E. Göransson, Anders Enocson

**Affiliations:** 1 Department of Trauma, Acute Surgery and Orthopaedics, Karolinska University Hospital, 171 64 Stockholm, Sweden; 2 Department of Medicine Solna, Karolinska Institutet, Stockholm, Sweden; 3 Department of Physiology and Pharmacology, Karolinska Institutet, Stockholm, Sweden; 4 School of Health and Welfare, Dalarna University, Falun, Sweden; 5 Department of Molecular Medicine and Surgery, Karolinska Institutet, Stockholm, Sweden

**Keywords:** Trauma, Chest injury, Thoracic trauma, Poly trauma, Mortality

## Abstract

**Background:**

Thoracic injuries are common among trauma patients. Studies on trauma patients with thoracic injuries have reported considerable differences in morbidity and mortality, and there is limited research on comparison between trauma patients with and without thoracic injuries, particularly in the Scandinavian population. Thoracic injuries in trauma patients should be identified early and need special attention since the differences in injury patterns among patient population are important as they entail different treatment regimens and influence patient outcomes. The aim of the study was to describe the epidemiology of trauma patients with and without thoracic injuries and its influence on 30-day mortality.

**Methods:**

Patients were identified through the Karolinska Trauma Register. The Abbreviated Injury Scale (AIS) system was used to find patients with thoracic injuries. Logistic regression analysis was performed to evaluate factors [age, gender, ASA class, GCS (Glasgow Coma Scale), NISS (New Injury Severity Score) and thoracic injury] associated with 30-day mortality.

**Results:**

A total of 2397 patients were included. Of those, 768 patients (32%) had a thoracic injury. The mean (± SD, range) age of all patients (n = 2397) was 46 (20, 18–98) years, and the majority (n = 1709, 71%) of the patients were males. There was a greater proportion of patients with rib fractures among older (≥ 60 years) patients, whereas younger patients had a higher proportion of injuries to the internal thoracic organs. The 30-day mortality was 11% (n = 87) in patients with thoracic injury and 4.3% (n = 71) in patients without. After multivariable adjustment, a thoracic injury was found to be associated with an increased risk of 30-day mortality (OR 1.9, 95% CI 1.3–3.0); as was age ≥ 60 years (OR 3.7, 95% CI 2.3–6.0), ASA class 3–4 (OR 2.3, 95% CI 1.4–3.6), GCS 1–8 (OR 21, 95% CI 13–33) and NISS > 15 (OR 4.2, 2.4–7.3).

**Conclusion:**

Thoracic injury was an independent predictor of 30-day mortality after adjustment for relevant key variables. We also found a difference in injury patterns with older patients having a higher proportion of rib fractures, whilst younger patients suffered more internal thoracic organ injuries.

## Introduction

Trauma is the leading cause of death and disability in people under the age of 45 years worldwide, and trauma related deaths outnumber even all cancer related deaths in young adults [1]. In severely injured trauma patients, thoracic injuries rank as important injuries and up to 50% of polytrauma patients endure some sort of thoracic injury [2, 3]. The mortality rate in polytrauma patients with thoracic injury varies in the previous literature [4–7]. The morbidity and mortality rates might be related to the thoracic injury itself, but there is limited information in the literature about its influence after adjustment of other relevant variables [8]. A thoracic injury can be caused by a blunt and/or a penetrating trauma [9]. In Sweden, blunt force trauma accounts for approximately 90% of all traumas [1]. Internationally, blunt force trauma against the chest has been reported to be the third leading cause of death, after head and abdominal injuries in conjunction with traffic accidents [10]. Injury to the thorax may affect the chest wall (rib and sternum fractures) as well as the thoracic organs (greater vessels, heart and lungs and pneumo- and haemothorax) [11]. Rib fractures are among the most common thoracic injury in patients following high-energy trauma [12]. The more rib fractures a patient sustains, the higher the mortality rate, usually due to additional underlying organ damage [13]. Various studies on trauma patients with thoracic injuries have indicated considerable differences in their morbidity and mortality, though there is limited research on comparison between trauma patients with and without thoracic injuries. [14, 15]

The aim of this study was to describe the epidemiology of trauma patients with and without thoracic injuries, and its influence on the risk of 30-day mortality.

## Patients and methods

### Study population

The Karolinska Trauma Centre (KTC) at the Karolinska University Hospital is the largest tertiary trauma care unit in Sweden, with approximately 1500 trauma alerts per year. It has a primary catchment area of approximately 2 million residents, and a secondary catchment area of approximately 3 million [16]. All patients arriving to the KTC as a primary trauma alert (or as a secondary transport to KTC after a trauma alert with primary survey at another hospital) are registered in the Karolinska Trauma Register (KTR). The KTR has, since 2002, served as an internal quality register for the Karolinska University Hospital with the purpose of improving trauma care throughout the whole treatment chain from pre-hospital management to definitive care. Variables in the KTR are registered according to the Utstein template [17]. The study period was January 1st, 2018, to December 31st, 2019. Patients with a thoracic injury were identified using the Abbreviated Injury Scale (AIS) coding system for injured body regions and type of injury [18]. The types of thoracic injuries were defined using the AIS codes. Variables collected for each patient were; patient characteristics (age, gender), ASA (American Society of Anesthesiologists) class and injury characteristics [ISS (Injury Severity Score) [19], NISS (New Injury Severity Score) [20], GCS (Glasgow Come Scale)] [21]. All patients 18 years old and above registered in the KTR were included in the study. Exclusion criteria were: dead upon arrival to the KTC, no registered AIS code, and several subsequent trauma alerts/registry for the same patient based on patient identification number (only the first registration for each patient was included in the analysis).

### Statistical methods

Categorical data was presented as frequency and percent distribution. Variables were tested using the Fisher’s exact test. Numerical data was presented as mean with ± SD (standard deviation) or median with IQR (interquartile range). The Mann–Whitney U-test was used for comparisons of independent groups. All tests were two-sided. Logistic regression analysis was performed to evaluate factors associated with 30-day mortality. Age (< 60 *versus* ≥ 60 years), gender (male *versus* female), ASA class (1–2 *versus* 3–4), GCS (1–8 including patients intubated prior to hospital arrival *versus* 9–15), NISS (1–15 *versus* > 15) and thoracic injury (yes *versus* no) were tested. First, crude associations for each variable were tested in univariable models. Second, a multivariable model was used to study the adjusted associations. The associations were presented as odds ratios (ORs) with 95% confidence intervals (CIs). The results were considered significant at *p* < 0.05. The statistical software used was IBM SPSS Statistics, version 25 for Windows (SPSS Inc., Chicago, Illinois).

## Results

**Fig. 1:**
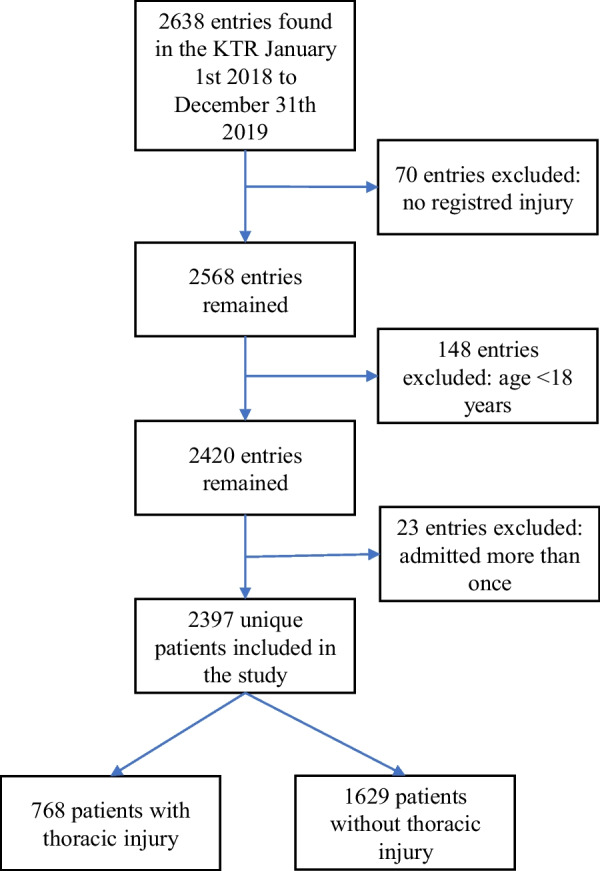
Flowchart of the inclusion of participants in the study. KTR = Karolinska Trauma Register

A total of 2638 trauma entries were identified in the KTR. After removal of 70 entries with no registered injury (no AIS code), 148 entries with patients < 18 years old and 23 secondary entries where patients were admitted more than once during the study period, 2397 unique patients with one trauma entry remained and were included in the study. Of those, 768 patients (32%) had a registered thoracic injury (Fig. 1).

### Epidemiology and injury patterns

The dominating type of injury was a blunt trauma (n = 2008, 84%) and the dominating injury mechanism was a high fall (n = 569, 24%), followed by a non-motorcycle related motor vehicle accident (n = 505, 21%) and a low fall (n = 323, 14%). A total of 300 (13%) patients were admitted due to a stabbing injury. Apart from the 768 patients that had a thoracic injury, the most common concurrent injury locations for all patients were; upper limb (n = 1013, 42%), head (n = 993, 41%) and lower limb (n = 991, 41%). Additional data on epidemiology and injury patterns are presented in Table 1.

**Table 1:** Epidemiology, vital signs, injury characteristics, hospital length of stay and mortality

Variable	All patients (n = 2397)	Patients with thoracic injury (n = 768)	Controls (patients without thoracic injury) (n = 1629)
Age; Mean (± SD, range)	46 (20, 18–98)	47 (19, 18–96)	46 (20, 18–98)
Age ≥ 60; n = (%)	620 (26)	212 (28)	408 (25)
Gender Female; n = (%)	688 (29)	176 (23)	512 (31)
^a^ASA-class 3–4; n = (%)	437 (18)	147 (19)	290 (18)
^*b*^*Injury mechanism; n* = *(%)*			
High fall	569 (24)	203 (26)	366 (23)
Low fall	323 (14)	45 (5.9)	278 (17)
MVA	505 (21)	196 (26)	309 (19)
MC	149 (6.2)	60 (7.7)	89 (5.5)
Bicycle	192 (8.0)	59 (7.6)	133 (8.2)
Gunshot	87 (3.6)	21 (2.8)	66 (4.0)
Stabbing	300 (13)	112 (15)	188 (12)
Other	258 (11)	69 (9.0)	189 (12)
*Type of injury; n* = *(%)*			
Blunt	2008 (84)	634 (83)	1374 (84)
Penetrating	389 (16)	134 (17)	255 (16)
ISS; Median (IQR)	9 (15)	14 (16)	5 (9)
ISS > 15; n = (%)	608 (25)	365 (48)	243 (15)
NISS; Median (IQR)	10 (19)	17 (20)	6 (14)
NISS > 15; n = (%)	880 (37)	476 (62)	404 (25)
^c^GCS; Median (IQR)	15 (0)	15 (0)	15 (0)
GCS < 9; n = (%)	88 (3.7)	25 (3.3)	63 (3.9)
^d^SBP (mmHg); Median (IQR)	136 (28)	135 (30)	137 (27)
Shock; n = (%)	96 (4.0)	71 (9.3)	25 (1.5)
Head injury; n = (%)	993 (41)	297 (39)	696 (43)
Face injury; n = (%)	887 (37)	268 (35)	619 (38)
Neck injury; n = (%)	302 (13)	89 (12)	213 (13)
Abdominal injury; n = (%)	418 (17)	216 (28)	202 (12)
Spine injury; n = (%)	482 (20)	257 (34)	225 (14)
Upper limb injury; n = (%)	1013 (42)	432 (56)	581 (36)
Lower limb injury; n = (%)	991 (41)	379 (49)	612 (38)
Hospital length of stay (days); Median (IQR)	2 (5)	4 (8)	2 (4)
^e^Mortality 30 days; n = (%)	158 (6.6)	87 (11)	71 (4.4)

*IQR* Interquartile Range, *ASA* American Society of Anesthesiologists, *MVA* Motor Vehicle Accident, *MC* Motorcycle, *ISS* Injury Severity Score, *NISS* New Injury Severity Score, *GCS* Glasgow Coma Scale, *SBP* Systolic Blood Pressure, *Shock* SBP < 90 mmHg

Missing data: ^a^ASA class n = 7, ^b^Injury mechanism n = 14, ^c^GCS n = 18, ^d^SBP n = 52 and ^e^Mortality 30 days n = 30 (all foreigners and therefore lost from follow-up)

### Injury severity and vital signs

The median (IQR) ISS and NISS was 9 (15) and 10 (19), respectively for all patients. The ISS (median, IQR) was higher for patients with thoracic injury (14, 16) compared to other patients (5, 9) (*p* < 0.001). Similarly, the NISS (median, IQR) was higher for patients with thoracic injury (17, 20) compared to other patients (6, 14) (*p* < 0.001).

The median (IQR) GCS on arrival was 15 (0) for all patients. A total of 227 (9.5%) patients (thoracic injury n = 125, 16%, other patients n = 102, 6.2%, *p* < 0.001) were intubated prior to arrival at hospital and thus had no adequate GCS value on arrival.

The median (IQR) systolic blood pressure (SBP) on arrival at hospital was 136 (28) mmHg for patients that had a measurable SBP. 71 (9.3%) of the patients with thoracic injury were in circulatory shock (SBP < 90 mmHg) on hospital arrival, compared to 25 (1.5%) among patients without thoracic injury (*p* < 0.001). Additional data on vital signs on arrival and injury severity are presented in Table 1.

### Thoracic injuries

In patients with thoracic injury (n = 768), the most common thoracic injury types were: rib fracture (n = 434, 57%), pneumothorax (n = 265, 35%) and hemothorax (n = 174, 23%) (Table 2). 166 patients received a chest drain. There was a greater proportion of patients with rib fractures amongst older (≥ 60 years) patients (n = 157, 74%) compared to younger patients (< 60 years) (n = 277, 50%) (*p* < 0.001).

**Table 2:** Type of thoracic injury in relation to age and gender, in patients with thoracic injury

Injury type	All (n = 768)*	Age (years)			Gender		
		< 60 (n = 556)	≥ 60 (n = 212)	p-value	Female (n = 176)	Male (n = 587)	p-value
Rib facture; n = (%)	434 (57)	277 (50)	157 (74)	< 0.001	109 (62)	325 (55)	0.1
Pneumothorax; n = (%)	265 (35)	207 (37)	58 (28)	0.01	60 (34)	205 (35)	0.9
Hemothorax; n = (%)	174 (23)	128 (23)	46 (22)	0.8	31 (18)	143 (24)	0.07
Lung contusion; n = (%)	140 (18)	113 (20)	27 (13)	0.02	38 (22)	102 (17)	0.2
Vascular injury; n = (%)	37 (4.8)	34 (6.1)	3 (1.4)	0.004	6 (3.4)	31 (5.3)	0.4
Heart injury; n = (%)	25 (3.3)	21 (3.8)	4 (1.9)	0.3	3 (1.7)	22 (3.7)	0.2

*Each patient could have several types of injuries

Younger patients had a higher proportion of pneumothorax (n = 207, 37% versus n = 58, 28%, *p* = 0.001), lung contusion (n = 113, 20% versus n = 27, 13%, *p* = 0.02) and vascular injury (n = 34, 6.1% versus n = 3, 1.4%, *p* = 0.004), compared to older (≥ 60 years) patients. No differences in the distribution of thoracic injuries were seen when comparing females with males (Table 2). Of the 434 patients with rib fractures, 122 patients (28%) had 1–2 fractures and 312 patients (72%) had ≥ 3 fractures.

### Mortality

The overall 30-day mortality was 6.6% (n = 158). There was an increased 30-day mortality in patients with thoracic injury (n = 87, 11%) compared to patients without (n = 71, 4.4%) (*p* < 0.001). To evaluate factors influencing the risk for 30-day mortality logistic regression analysis was performed. After multivariable adjustment, thoracic injury was found to be associated with an increased risk of 30-day mortality (OR 1.9, 95% CI 1.3–3.0), as was age ≥ 60 years (OR 3.7, 95% CI 2.3–6.0), ASA class 3–4 (OR 2.3, 95% CI 1.4–3.6), GCS 1–8 (OR 21, 95% CI 13–33) and NISS > 15 (OR 4.2, 2.4–7.3) (Table 3).

**Table 3:** Logistic regression to evaluate factors associated with 30-day mortality

	30-day mortality	Univariable		Multivariable	
	n (%)	OR (95%CI)	p-value	OR (95%CI)	p-value
*Age*					
< 60 years	73 (4.1)	1 (reference)		1 (reference)	
≥ 60 years	85 (14)	3.7 (2.7- 5.1)	< 0.001	3.7 (2.3–6.0)	< 0.001
*Gender*					
Male	113 (6.7)	1 (reference)		1 (reference)	
Female	45 (6.6)	1.0 (0.7- 1.4)	0.9	1.1 (0.7–1.7)	0.7
*ASA*					
1–2	83 (4.3)	1 (reference)		1 (reference)	
3–4	69 (16)	4.2 (3.0–5.9)	< 0.001	2.3 (1.4–3.6)	< 0.001
*GCS*					
9–15	40 (1.9)	1 (reference)		1 (reference)	
1-8^a^	115 (37)	30 (20–44)	< 0.001	21 (13–33)	< 0.001
*NISS*					
1–15	20 (1.3)	1 (reference)		1 (reference)	
> 15	138 (16)	14 (8.8–23)	< 0.001	4.2 (2.4–7.3)	< 0.001
*Thoracic injury*					
No	71 (4.3)	1 (reference)		1 (reference)	
Yes	87 (12)	2.9 (2.0–3.9)	< 0.001	1.9 (1.3–3.0)	0.002

*OR* Odds Ratio, *CI* Confidence Interval, *ASA* American Society of Anesthesiologists, *GCS* Glasgow Coma Scale, *NISS* New Injury Severity Score

^a^Includes patients intubated prior to hospital arrival

## Discussion

We found that thoracic injuries were common, affecting about one third of all trauma patients, and that the typical patient was a middle-aged male who had suffered a blunt trauma. Our main finding was that thoracic injuries in trauma patients were found to be associated with an increased risk of 30-day mortality after adjustment of key variables. This finding resembles other studies and shows that a thoracic injury is an independent predictor of mortality in the trauma patients, irrespective of the severity of the trauma. In a seven-yearlong study by Grubmüller et al., they demonstrated that trauma patients with a thoracic injury had a mortality rate of 13%, without any noteworthy difference between mild or severe thoracic trauma. Their slightly higher mortality compared to ours (11%) can possibly be explained by the fact that they only included patients with ISS > 16 [22]. In a study from 2017 investigating changes in mortality over time, Horst et al., concluded that *predicted yearly death rates* for patients with thoracic injuries had improved and was 4.7% at the end of their study period. The authors suggested that this was because diagnostic and treatment had improved over time [23]. Gaillard et al., included 1026 trauma patients during 5.5 years. They reported that the mortality rate increased significantly if the trauma patients had sustained some sort of chest lesion, with a flail chest being the most severe type of injury associated with 69% mortality during the study period [24]. Also, Platz et al., correlated different types of thoracic injuries with different rates of mortality and morbidity. They reported that an aortic injury had a mortality of 31%, an oesophageal injury up to 40% and a tracheobronchial tree disruption was rare but had a mortality rate of 80% [25]. Heus et al. [26] revealed in a nine-yearlong study that mortality rate was 7.5% for patients with penetrating thoracic injuries and the most common injury then was a pneumothorax. These findings were similar to a recent article by Nyberger et al., [27] that reported a 30-day mortality for firearm injuries of 17% and that chest injuries (13%) were the fourth most common anatomical region affected. The most common anatomical region injured was the lower extremity (30%), followed by the upper extremity (14%). However, we think that mortality analyses on a very detailed level (specifically injured thoracic organ, injury mechanism, etc.) are hard to interpret due to the multifactorial causes of death in trauma patients and thus should be interpreted with caution.

It is well known that head injury is the leading cause of death in trauma patients [28–33]. In addition, our results support the assumption that head injury in combination with thoracic injury could be even worse for a patient and increase the risk for mortality. Also, we found differences in mortality with regards to co-morbidity (ASA class 1–2 compared to ASA 3–4). This is not surprising and is somewhat in line with our finding that old age (≥ 60 years) also was associated with an increased risk for 30-day mortality, because younger trauma patients can be expected to have fewer co-morbidities compared to older patients [34]. One can argue about our decision to use an age cut-off of 60 years, but this is an often-used level with regards to trauma outcomes and survival that has been used in several other studies [35–37].

In regard to the type of thoracic injury, rib fractures were the most common type of injury (58%). Similar findings have been presented by Zanette et al., [38] who found that rib fractures were present in 42% of all the patients with thoracic injury admitted to their hospital. In a recent (2022) nationwide study from the Netherlands, Peek et al., [39] found that 62% of all trauma patients sustained a thoracic injury and the most common (76%) type of thoracic injury were rib fractures. Another key finding in our study was that elderly trauma patients had more rib fractures than younger trauma patients, whereas the younger population was more prone to internal thoracic injuries including pneumothoraxes, lung and cardiac contusions. These differences in injury patterns are important as they require different treatment regimes. Internal injuries generally require interventional procedures such as chest tubes, coiling for bleeding, or in the unstable patient, a thoracotomy [40], whereas rib fractures, with few exceptions, are treated conservatively. In addition, elderly patients with rib fractures often require more nursing care and are more prone to multi-organ failure and hospital-acquired diseases, and in general require longer periods of convalescence [39, 40]. In addition, elderly patients often have underlying risk factors, such as osteoporosis, and thus can be poly-traumatized even after a low-energy trauma [39–42].

The typical trauma patient in our study was a middle-aged male who had suffered a blunt trauma. Overall, these findings on age and gender distribution were in accordance with other epidemiological studies in unselected trauma populations [24, 38, 43, 44]. Also, blunt trauma has been reported as the most common trauma mechanism in several other studies with comparable populations. [37, 40, 43]

### Strengths and limitations

The major strength of this study was the large sample size of an unselected trauma population. All trauma data was well documented due to the Swedish personal identification number and a reliable electronic journal system. Limitations included its retrospective design, and that the study only included data from one trauma centre, which may not be representative of an entire population. We also lacked clinical outcomes and long-term follow-up of mortality.

## Conclusion

A traumatic thoracic injury was an independent predictor of 30-day mortality after adjustment for several relevant key variables. In addition, we found a difference in injury patterns with older patients being more prone to having rib fractures and younger patients having more injuries in internal thoracic organs and structures.

## Data Availability

In accordance with ethical legislation data cannot be distributed to others.
